# Ubiquitin-specific protease 2 decreases p53-dependent apoptosis in cutaneous T-cell lymphoma

**DOI:** 10.18632/oncotarget.10268

**Published:** 2016-06-24

**Authors:** Tianling Wei, Edyta Biskup, Lise Mette Rahbek Gjerdrum, Omid Niazi, Niels Ødum, Robert Gniadecki

**Affiliations:** ^1^ Department of Dermatology, Bispebjerg Hospital, Copenhagen, Denmark; ^2^ Department of Pathology, Zealand University Hospital, Køge, Denmark; ^3^ Institute of Medical Microbiology and Immunology, University of Copenhagen, Copenhagen, Denmark; ^4^ Faculty of Health Sciences, University of Copenhagen, Copenhagen, Denmark

**Keywords:** ubiquitin-specific protease 2, cutaneous T-cell lymphoma, PUVA

## Abstract

Treatment of advanced cutaneous T-cell lymphomas (CTCL) is challenging because they are resistant to conventional chemotherapy. USP2 has been shown to promote resistance to chemotherapeutic agents in several cancer models.

We show here USP2 is expressed in quiescent and activated T-cells and its expression is 50% lower in CTCL cell lines (MyLa2000, SeAx and Hut-78) than in normal T-cells. USP2 is expressed in neoplastic cells in early, plaque-stage mycosis fungoides (MF) and is downregulated in advanced tumor stages. Upon treatment with psoralen with UVA (PUVA) or a p53 activator, nutlin3a, USP2 expression is significantly increased in MyLa2000 (p53^wt/wt^), but not in SeAx (p53^mut^) or Hut-78 (p53−/−). USP2 knockdown decreases MyLa2000 cell viability after PUVA by 50% but not Hut-78, suggesting that the function of USP2 in CTCL cells is p53-dependent. Furthermore, USP2 knockdown results in a decreased Mdm2 expression and upregulation of p53. Taken together, our findings suggest that USP2 stabilizes Mdm2 which antagonizes pro-apoptotic activity of p53 and possibly contributes to therapeutic resistance in CTCL.

## INTRODUCTION

Cutaneous T-cell lymphomas (CTCL) are extra-nodal lymphomas arising from skin-homing T-lymphocytes. Mycosis fungoides (MF) and Sézary syndrome (SS) are the most common entities. MF typically presents as skin patches and/or plaques, which can progress to skin tumours, with subsequent involvement of lymph nodes, peripheral blood, and visceral organs [1, 2]. In early stages, the disease can be controlled by skin-directed therapies such as psoralen with UVA photochemotherapy (PUVA), ionizing radiation, topical chemotherapy or topical corticosteroids. In the advanced, aggressive stages, CTCL is an incurable disease in which the 5-year mortality exceeds 50% [3, 4].

Deubiquitinating enzymes (DUBs) are known as one of the largest protein family which regulates the ubiquitin–proteasome system [5]. Ubiquitin-specific proteases (USP) comprise the largest subfamily in DUBs with more than 60 members. USPs counteracts proteasome degradation of proteins by deconjugating ubiquitin from substrates and from ubiquitin chains [6].

Alterations in ubiquitin-proteasome system are observed in the pathogenesis of many human diseases, including cancer [7], inflammatory and autoimmune diseases [8] and viral diseases [9]. The proteasome inhibitor bortezomib is approved for the treatment of multiple myeloma and mantle cell lymphoma, underscoring the hypothesis that the ubiquitin–proteasome system can be therapeutically targeted in cancer. Although bortezomib showed effect in CTCL, the dose-limiting toxicities and drug-resistance restrict the use of this drug [10, 11]. A promising alternative to targeting the proteasome itself would be to interact with the USPs to generate more specific, less toxic anticancer agents [12].

It has been previously shown that USP2 has oncogenic properties. USP2a is overexpressed in human prostate adenocarcinomas, and upregulation of USP2 prevents cancer cells from apoptosis upon treatment with chemotherapeutic agents [13–15]. Moreover, USP2 has been shown to restrain proteasomal degradation of specific proteins involved in different cellular pathways, such as Mouse double minute 2 homolog (Mdm2), MdmX, fatty acid synthase (FASN), apoptosis inducing factor (AIF), cyclin D, Aurora-A kinase and epidermal growth factor receptor (EGFR) [16–22]. The function of USP2 in CTCL has not been studied. Here, we found that USP2 showed decreased expression in advanced CTCL compared with the early stage disease and blocked apoptosis in malignant p53^wt^ T-lymphocytes. We propose that a negative regulatory loop in which USP2 is induced by PUVA via p53 increases cell resistance to apoptosis via deubiquitination of Mdm2.

## RESULTS

### USP2 is expressed in CTCL

**Figure 1:**
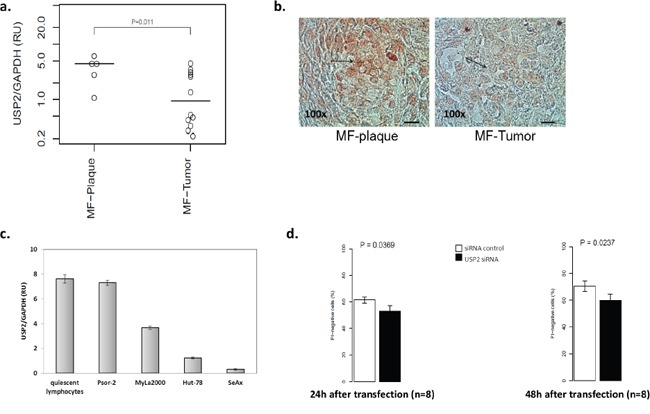
USP2 is expressed in CTCL with a stage-dependant manner Analysis of USP2 mRNA expression in paraffin-embedded biopsies from MF-plaque (n=5) and MF-tumor (n=13) **a.** and in quiescent lymphocytes and a panel of cell lines, Psor-2, MyLa2000, Hut-78 and SeAx **c.** by qPCR. Data was normalized to GAPDH and expressed as relative units (RU). Unpaired T-test was used to calculate P-value. Columns, mean (n = 3); error-bars, SD. **b.** USP2 protein expression in 5 MF-plaque and MF-tumor was detected by immunohistochemistry. Red color indicates USP2 expression. Bar=50μm. Arrows highlight lymphoma cells. **d.** Analysis of USP2 knockdown in MyLa2000 cell viability. Columns, mean (n = 8); paired T-test was used to calculate P-value, error-bars, SEM. PI-negative cells represent viable cells.

Using qPCR, the expression of the three known USP2 isoforms was determined in the material from paraffin-embedded biopsies from plaque/patch and tumor MF [23]. The expression of USP2b and UBP41 was not detected (data not shown). USP2a was expressed in both patch/plaque MF and tumor MF with 2.3 fold lower expression in the tumors (Figure 1a). In addition, we confirmed the presence of the protein by immunohistochemistry in the corresponding paraffin-embedded samples (Figure 1b). USP2 was detectable in keratinocytes, fibroblasts, and dermal T-cells. There were no obvious differences in USP2 immunoreactivitivity in keratinocytes and fibroblasts between the plaques and the tumors of MF (data not shown). However stronger USP2 staining was observed in lymphoma cells in the plaques than in the tumors (Figure 1b). Moreover, we determined the expression of USP2 in quiescent lymphocytes from healthy individuals (n=3), a T-cell cell line from a psoriasis patient (Psor-2), representing activated T-lymphocytes [24] and a panel of CTCL cell lines, MyLa2000 cells, SeAx and Hut-78 cells. The highest USP2 expression was detected in quiescent T-cells and Psor-2 and 50% lower USP2 expression was seen in MyLa2000 cells. The downregulation was even more pronounced in Hut-78 and SeAx cells (Figure 1c). The finding that USP2 was decreased in CTCL comparing to normal T-cells suggested that USP2 had a pro-apoptotic property. Therefore we investigated proliferation and viability in MyLa2000 cells after silencing of USP2 with siRNA. The efficiency of the transfection was confirmed by qPCR (Figure S1). MyLa2000 cells proliferation was not changed (Figure S2) Surprisingly, USP2 knockdown reduced viability of MyLa2000 cells (Figure 1d).

### USP2 is upregulated by PUVA and a p53 activator, nutlin3a

**Figure 2:**
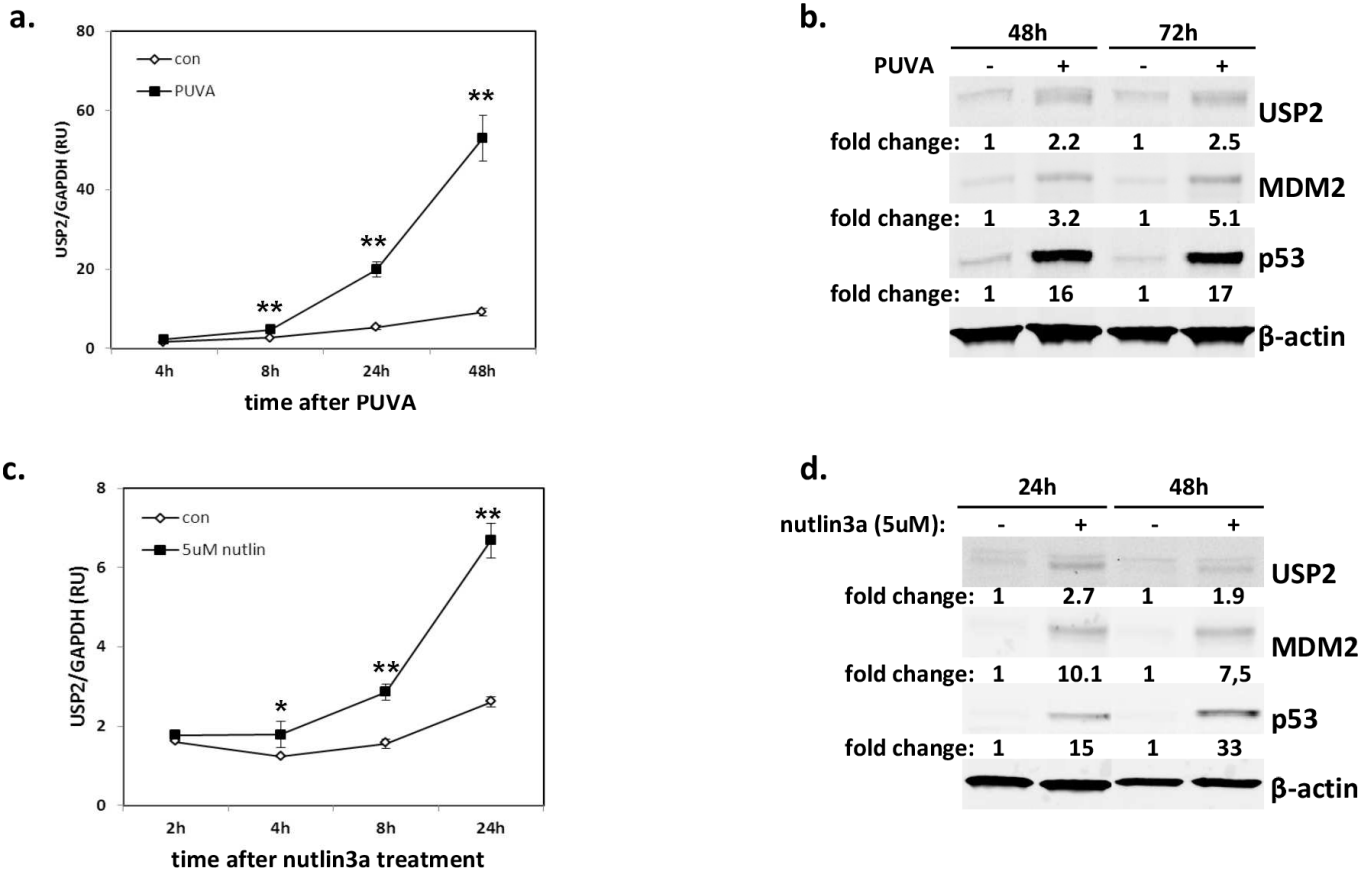
USP2 is induced by PUVA and a p53 activator, nutlin3a p53^wt^ CTCL cell line, MyLa2000, was subjected to PUVA **a, b.** or 5μM nutlin3a **c-d.** as shown in Methods. USP2 expression was measured by qPCR (a, c) and western blot (WB) (b, d) 2h-72h after the treatments. For qPCR, data was normalized to GAPDH and expressed as relative units (RU). The experiments were repeated 3 times, unpaired T-test was used to calculate P-value, error-bars, SD, *, P<0.05; **, P<0.01. For WB, Mdm2 and p53 protein level was also measured. β-actin was used as internal control, and relative protein expression levels are reported below the corresponding western blot bands. Representative data was shown.

Since PUVA is a first-line therapy for MF [25], we aimed to determine whether USP2 reduced cell sensitivity to this treatment. The kinetics of USP2 expression was examined in MyLa2000 cells for 4 to 72 hours after PUVA treatment. PUVA caused a significant (P<0.01) upregulation of USP2 mRNA expression already after 8 hours with an even stronger increase after 24 and 48 hours post-treatment (Figure 2a). USP2 protein expression was increased 2.2 fold and 2.5 fold 48h and 72h after PUVA, respectively. Mdm2 and p53 were also induced by PUVA (Figure 2b).

P53 is a major pro-apoptotic protein and unlike in many cancers it is rarely mutated in early MF [26–29]. Nutlin-3a is a known activator of p53 acting via disruption of p53-Mdm2 interaction and causes apoptosis in MyLa2000 which has intact p53, but not in SeAx and Hut-78 which have mutated p53 [29, 30]. Nutlin3a treatment caused a significant upregulation of p53 and Mdm2 protein expression in MyLa2000 cells (Figure 2d) accompanied by the upregulation of p53 target, p21 and Mdm2 (Figure S3). Nutlin 3a also caused an increase in USP2a mRNA and a 2.7-fold and 1.9-fold increase in USP2 protein expression 24h and 48h after treatment, respectively (Figure 2c, 2d).

### Knockdown of USP2 sensitizes MyLa2000 cells to PUVA and nutlin3a treatment

**Figure 3:**
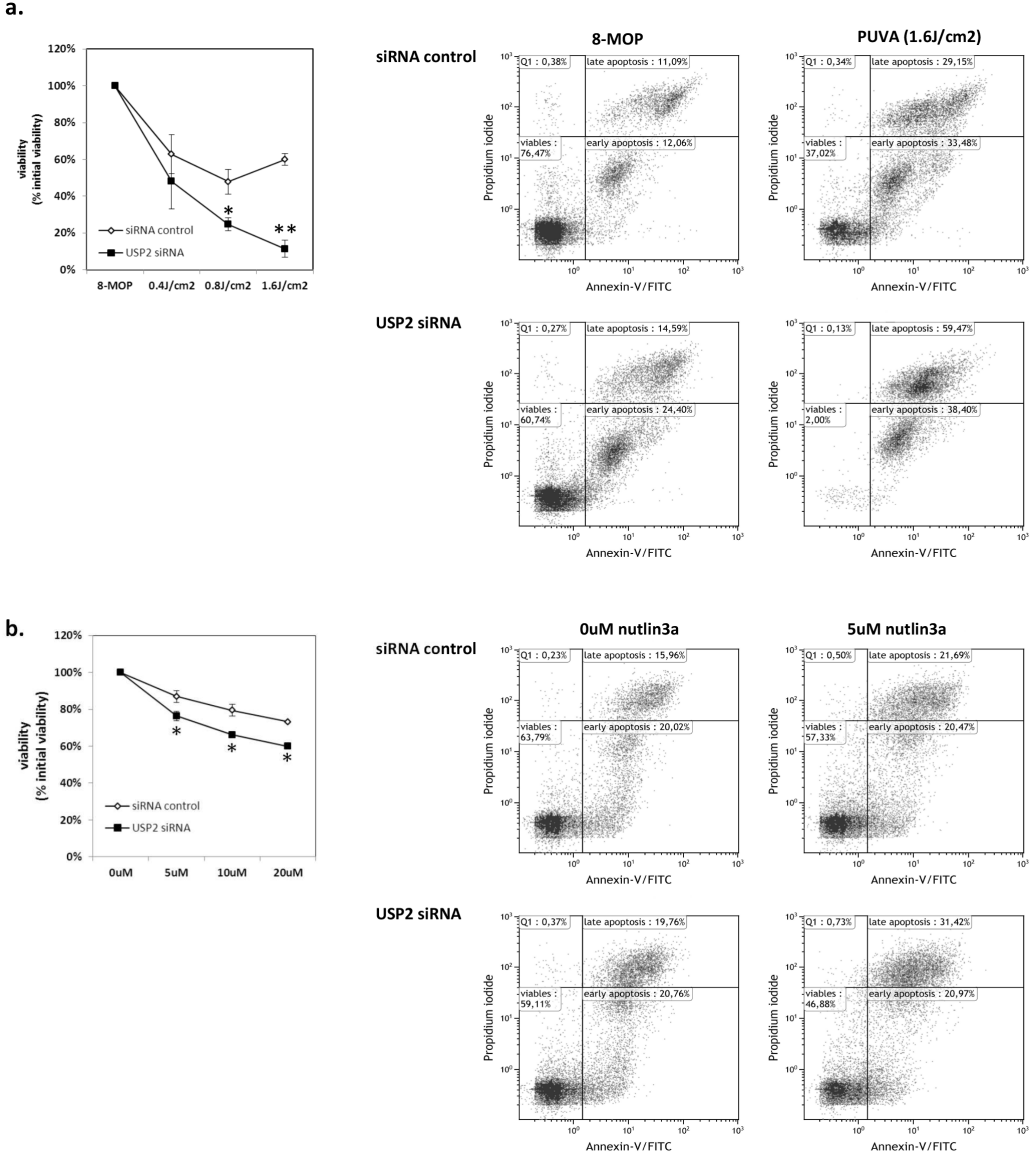
USP2 silencing promotes apoptosis in MyLa2000 cells upon PUVA or nutlin3a treatment USP2 knockdown enhances MyLa2000 apoptosis upon PUVA **a.** and nutlin3a **b.** treatment. MyLa2000 cells were transfected with USP2 siRNA or siRNA control. After 24h the cells were treated with the indicated dosage of PUVA and nutlin3a. 24h post nutlin3a and 48h post PUVA stimulation, cells were staining with annexin V and PI for flow cytometry, as described in Methods. Left: comparison of cell viability between cells with USP2 siRNA and siRNA control after treatment. Values are means of three independent experiments, paired T-test was used to calculate P-value, Error-bars, SEM. *, P<0.05; **, P<0.01. Right: The dot-plot graphs are representative of three independent experiments.

USP2 was downregulated in advanced stage CTCL and induced by PUVA and p53 activator, nutlin3a, suggesting that USP2 acted as a survival stress response to prevent lymphoma cells from apoptosis. Therefore we investigated whether apoptosis caused by PUVA or nutlin3a in MyLa2000 cells can be aggravated by knockdown of USP2. The efficiency of transfection was confirmed by qPCR (Figure S1). Silencing of USP2 sensitized MyLa2000 cells to apoptosis after treatment with PUVA or nutlin3a (Figure 3a, 3b).

### USP2 reduces cell apoptosis via p53 signaling

**Figure 4:**
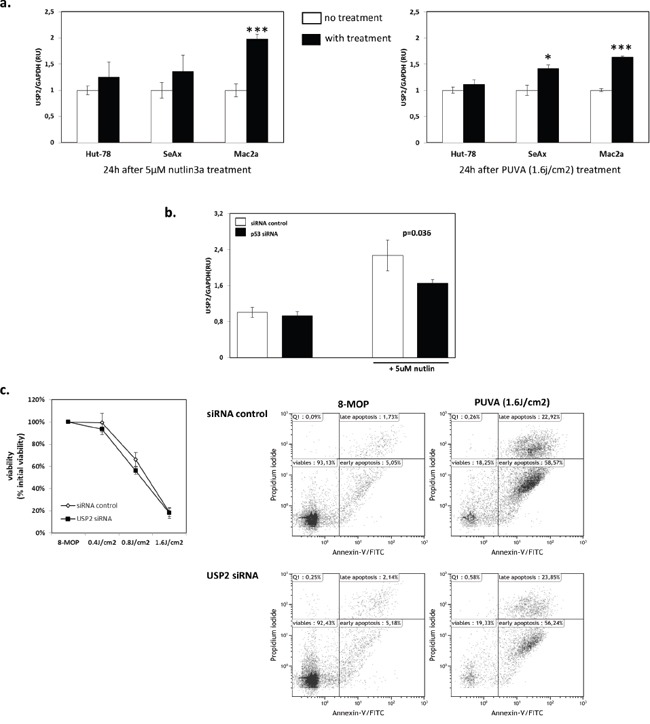
The upregulation of USP2 is p53-dependent **a.** USP2 expression is induced by nutlin3a and PUVA in Mac2a with functional p53, but not in Hut-78 and SeAx cells with impaired p53. USP2 expression was examined in Hut-78, SeAx and Mac2a cells treated with nutlin3a and PUVA as shown in figure 2. The experiment was repeated 3 times. Unpaired T-test was used to calculate P-value, error-bars, SD. **b.** Knockdown of p53 reduces induction of USP2 expression upon nutlin3a treatment. USP2 expression was checked in MyLa2000 cells with silenced p53 24h after nutlin3a treatment. Values are means of three independent experiments, paired T-test was used to calculate P-value, Error-bars, SEM. **c.** Hut-78 apoptosis is not increased upon USP2 knockdown after PUVA treatment. The experiment was performed and presented as described in figure 3. Paired T-test was used to calculate P-value, Error-bars, SEM.

To further confirm that p53 signaling was involved in the regulation of USP2, we investigated whether USP2 was inducedby nutlin3a or PUVA in the other CTCL cell lines with functional p53 signaling (Mac2a) and impaired p53 signaling, SeAx (p53^mut^) and Hut-78 (p53^−/−^) [29]. USP2 mRNA expression was increased in Mac2a upon both treatments, indicating that the upregulation of USP2 was not MyLa2000 cells-specific (Figure 4a). As expected, USP2 mRNA expression was not increased by 5uM nutlin3a in SeAx or Hut-78 (Figure 4a). More importantly, PUVA did not upregulate USP2 mRNA expression in Hut-78 cells and only slightly in SeAx cells (Figure 4a). Moreover, the effect of nutlin3a in USP2 mRNA expression was examined in MyLa2000 cells with knockdown of p53. USP2 was induced in MyLa2000 cells. However, the induction of USP2 was significantly lower in MyLa2000 with silenced p53 (Figure 4b). Knockdown of USP2 did not increase cell apoptosis in Hut-78 cells compared to cells with control siRNA, further suggesting the pivotal role of p53 in USP2 function (Figure 4c).

### Knockdown of USP2 decreases Mdm2 protein level and increases p53 transcriptional activity

**Figure 5:**
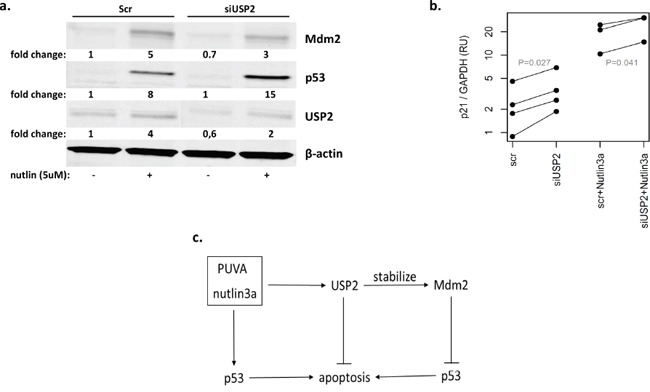
USP2 inhibits the cellular actions of p53 through Mdm2 **a.** USP2 knockdown decreased Mdm2 expression. MyLa2000 cells were transfected with either siRNA control or USP2 siRNA for 24h, followed by treatment with 5μM nutlin3a for 24h. Western blot was used for detecting USP2, Mdm2 and p53 protein level. β-actin was used as internal control, and relative protein expression levels are reported below the corresponding western blot bands. The experiment was repeated 3 times. Representative data was shown. **b.** USP2 knockdown increase p53 transcription activity. The expression of p21 was used to indicate p53 transcription activity. qPCR was applied to examine p21 expression in MyLa2000 cells transfected with siUSP2/siRNA with or without nutlin3a treatment. Paired T-test was used to calculate P-value. **c.** A proposed model for the regulation of USP2. PUVA and nutlin3a activate p53 and promote cell apoptosis. USP2 is induced by PUVA and nutlin3a and increases cell resistance to apoptosis via modulation of Mdm2/p53 interaction, constituting an anti-apoptosis protective loop.

It was previously shown that USP2 stabilized Mdm2 in NTERA-2 testicular embryonal carcinoma cells, which express wild-type p53 and Mdm [17]. To test whether USP2 could block p53 via Mdm2, we silenced USP2 and measured Mdm2 level by western blot in MyLa2000 cells after nutlin3a treatment. USP2 knockdown resulted in 30% and 40% lower Mdm2 protein expression in nutlin3a-nontreated and treated cells, respectively. The expression of p53, was increased by nutlin3a in cells with USP2 knockdown to a higher extent than in the control MyLa2000 cells treated by scrambled oligonucleotide (Figure 5a). In addition, we also investigated whether the knockdown of USP2 and nutlin3a in combination increased p53 transcriptional activity. The upregulation of p21 was used to indicate the increased p53 transcriptional activity. As shown in Figure 5b, USP2 knockdown significantly increased p21 mRNA expression compared with MyLa2000 cells with control siRNA (p<0.05) (Figure 5b). These results suggested that activated p53 increased USP2, which in turn counteracted the pro-apoptotic activity of p53, forming a negative feedback loop (Figure 5c).

## DISCUSSION

Modification of the ubiquitin-proteasome system has emerged as a strategy for cancer treatment [6, 31]. In this study, we show for the first time the function of USP2a in CTCL. USP2a has been investigated in many types of cancers, including prostate cancer [15], gliomas [32], ovarian serous cystadenocarcinoma [33] and bladder cancer [14] where its expression correlated with cancer grade with higher expression in advance stages. Surprisingly, expression of USP2a was highest in normal T-lymphocytes and further decreased in advanced CTCL. These results implicated that unlike the situation seen in solid tumors, USP2a may be a tumor suppressor in CTCL. However, we refuted this hypothesis by showing that a knockdown of USP2a promoted apoptosis of MyLa2000 cells. Moreover, USP2 silencing enhanced apoptosis after PUVA treatment in MyLa2000 suggesting that USP2 enhanced therapeutic resistance. It would be interesting to investigate whether USP2 aggravates the resistance to other therapeutic modalities, such as ionizing radiation or ultraviolet B.

Mechanistically we were able to demonstrate that the activation of p53 upregulated USP2. First, a selective p53 activator nutlin3a increased USP2a in p53^wt^ CTCL cell lines, MyLa2000 and Mac2a, but not in p53 impaired CTCL cell lines. Second, the increase of USP2a after PUVA was absent or largely diminished in the cell lines with mutated p53 (Hut-78 and SeAx). A slight increase of USP2 expression in SeAx cells after PUVA is compatible with the residual activity of mutated p53 in this cell line. Third, the induction of USP2 in MyLa2000 cells was significantly lower upon knockdown of p53 after treatment of nutlin3a. Finally, the knockdown of USP2 in Hut-78 cells did not increase apoptosis after PUVA, in contrast to what was observed in MyLa2000 with intact p53.

Although we were able to demonstrate that USP2 stabilized Mdm2, we do not have direct evidence to postulate that this is due to the changes in deubiquitination of the protein. A direct measurement of the levels of ubiquitinated Mdm2 should be performed to unequivocally link USP2 deubiquitinase activity with the Mdm2-p53 signaling.

Taken together, we show for the first time that USP2 may be anti-apoptotic in CTCL and antagonize the effect of PUVA. We postulate that upregulation of USP2 antagonizes p53 effect via deubiquitination of Mdm2. In the future, targeting USP2 may be utilized in the therapy of CTCL.

## MATERIALS AND METHODS

### Clinical samples, cell culture and reagents

Formalin-fixed, paraffin-embedded biopsies taken from 5 patients with the MF plaques (Stage IA-IB) (3 males and 2 females; mean age 68 years; range 49 to 87 years) and 13 MF tumors (Stage IIB) (7 males and 6 females; mean age 65 years; range 47 to 91 years) were obtained from the Department of pathology, Rigshospitalet. Four CTCL cell lines were used: MyLa2000, derived from a plaque biopsy of a patient with MF [34], SeAx [35] and Hut-78 [36], derived from peripheral blood of patients with SS, Mac2a (Davis et al., 1992), derived from different clinical specimens of a patient showing progression from lymphomatoid papulosis to anaplastic large-cell lymphoma. Cells were maintained as described [37, 38]. Briefly, MyLa2000 and SeAx cells were cultured in Gibco GlutaMAX (Invitrogen, Carlsbad, CA) supplemented with 10% fetal bovine serum at 37°C under 5% CO2. Hut-78 and Mac2a cells were cultured in RPMI-1640 containing 2 mm l-glutamine and 10% fetal bovine serum. Psor-2 was isolated from involved skin of a psoriatic donor [24] and were maintained in RPMI-1640 containing 2 mm l-glutamine and 10% human serum. Nutlin-3a was obtained from Cayman Chemical (Ann Arbor, MI), and methoxsalen, also known as 8-MOP, was purchased from Sigma-Aldrich (St. Louis, MO).

For PUVA, cells were incubated with 1μM methoxsalen (8-MOP) for 2h prior to 0.4, 0.8 or 1.6 J/cm^2^ UVA exposure. Control was cells treated with 8-MOP, but no UVA irradiation.

### siRNA transfections

For both MyLa2000 and Hut-78 cells, transfection was carried out using Amaxa machine (Lonza, Basel, Switzerland) and Nucleofector Kit-T (Cat VCA-1002) according to manufacturer's instruction. Briefly, 10×10^6^ cells were centrifuged at 90g for 6 minutes and resuspended in 100 μl Nucleofector® Solution combined with siNRAs at room temperature. 50 nM small interfering RNA was used for the specific knockdown of USP2 (Santa Cruz Biotechnology, Texas, USA) and p53 (Thermo Scientific, Chicago, USA). Silencer select negative control #1 (Ambion Inc., Austin, TX, USA) was used as negative control. Cell/siRNA suspension was transferred into certified cuvette, and Nucleofector® Program T-016 was chose for MyLa2000 cells and V-001 was used for Hut-78 cells. Efficiency of transfection was evaluated at mRNA level by RT-PCR (Figure S1) and at protein level by western blot analysis.

### RNA extraction

Total RNA from paraffin-embedded blocks was extracted by applying RNeasy FFPE Kit (Qiagen, Venlo, Limburg, Netherlands) according to manufacturer's instruction. Briefly, 10μm thick sections were cut from FFFE blocks and 6 sections from each block were placed into a microcentrifuge tube for RNA extraction. The sections were treated with 320μl deparaffinization buffer (Qiagen) and 240μl buffer PKD. The customized protocol, RNeasy FFPE - 3-8 FFPE tissue sections – Standard, was selected to perform RNA extraction in QIAcube (Qiagen).

Total RNA from cells was prepared using QIAcube. The protocol, RNeasy Mini - Animal tissues and cells – Standard, was applied to extract RNA. The RNA concentration was determined by spectrophotometry. Total RNA (0.5μg) was reversed transcribed into cDNA in a 20μl reaction by RevertAid™ First Strand cDNA Synthesis Kit (Thermo Scientific). The resulting cDNA was diluted with 80μl dH_2_O to obtain a concentration of 5 ng/μl cDNA.

### Quantitative real-time PCR

For the quantification of Mdm2, USP2 and p21, 20 ng of cDNA per reaction were amplified in the presence of TaqManR universal master mix (Applied Biosystems, Foster City, CA) and TaqMan® Gene Expression Assay: Mdm2 (Hs01066930-m1), USP2 (Hs00899199-g1), p21 (Hs00355782-m1), GAPDH (Hs02758991_g1)(Applied Biosystems) (stage 1, 50°C for 2 min, stage 2, 95°C for 10 min and stage 3, 95°C for 15 s, 60°C for 1 min, repeated 45 times) in Stratagene Mx3005p (Aglicent Technologies, Santa Clara, CA). Target gene expression was normalized based on the values of the expression of GAPDH (Applied Biosystems).

### Apoptosis assessment

In order to assess the apoptotic rate, MyLa2000 cells and Hut-78 were transfected with USP2 siRNA and control siRNA, followed by treatment with PUVA or nutlin3a. 24h or 48h after treatment, cells were double-stained with annexin V-FITC and PI according to the manufacturer's protocol (Beckman-Coulter, Fullerton, CA) and analyzed by flow cytometry (Beckman-Coulter, Fullerton, CA) as described previously [39, 40].

### Western blotting and immunohistochemistry

MyLa2000 cell were lysed for protein expression by western blotting. The protein concentration was determined by Pierce™ BCA Protein Assay Kit (Thermo Scientific) according to manufacturer's instruction. The primary antibodies used were USP2 (8036s diluted 1:500) from Cell Signaling (Danvers, Massachusetts, USA), anti-Mdm2 (33-7100 diluted 1:500) from Invitrogen (Carlsbad, CA) and anti-p53 (M3629 diluted 1:500) from DAKO (Glostrup, Denmark). Equal amounts of protein were loaded in a 4-12% Bis-Tris gel (Bio-Rad Laboratories, Hercules, CA) and separated by electrophoresis at 200 V followed by electrophoretic transfer to a nitrocellulose membrane (Bio-Rad Laboratories). Membranes were then blocked with Odyssey^®^ blocking agent (Li-Cor, Lincoln, NE) for 1 hour at 4°C. Primary mouse or rabbit antibodies were applied overnight at 4°C followed by incubation with the appropriate secondary antibodies DyLight 800 conjugate (anti-rabbit) (Thermo Scientific) or Alexa Fluor 680 (anti-mouse) (Invitrogen Cooperation, Carlsbad, CA) for 2h at room temperature. Expression was detected and quantified with the infrared Odyssey imaging System (Li-Cor). USP2 protein expression was analysed in formalin-fixed paraffin-embedded skin sections using rabbit anti-human USP2 antibody (ab101609 diluted 1:100) from Abcam (Cambridge, UK) and anti-rabbit conjugated with HRP (P 0448) (DAKO) at 1: 200 dilution. Sections were mounted using Glycergel® Mounting Medium, Aqueous (DAKO). The omission of the primary antibody in the staining procedure was used as a negative control.

### Statistics

Two-sided heteroscedastic Student's t-test was used for two-group comparisons of continuous data, Paired or unpaired as indicated in the figure caption. A p-value less than 0.05 was considered statistically significant. Continuous data are reported as means with standard deviation (SD) or standard error of the mean (SEM) as noted in figure caption. Real Time-PCR data were analyzed using the ΔΔCT method. Statistical analysis was performed by R (version 3.1.0, Vienna, Austria) or Excel (Microsoft Corp., Redmond, WA).

## SUPPLEMENTARY FIGURES


