# Supplementary material for: Ubiquitin-specific protease 2 decreases p53-dependent apoptosis in cutaneous T-cell lymphoma
Source: Oncotarget. 2016 Jun 24;7(30):48391–400. doi: 10.18632/oncotarget.10268 (PMC5217025; doi:10.18632/oncotarget.10268)
Supplement: Supplementary file 1 [file oncotarget-07-48391-s001.pdf]

Ubiquitin-specific protease 2 decreases p53-dependent apoptosis in cutaneous T-cell lymphoma

SUPPLEMENTARY FIGURES

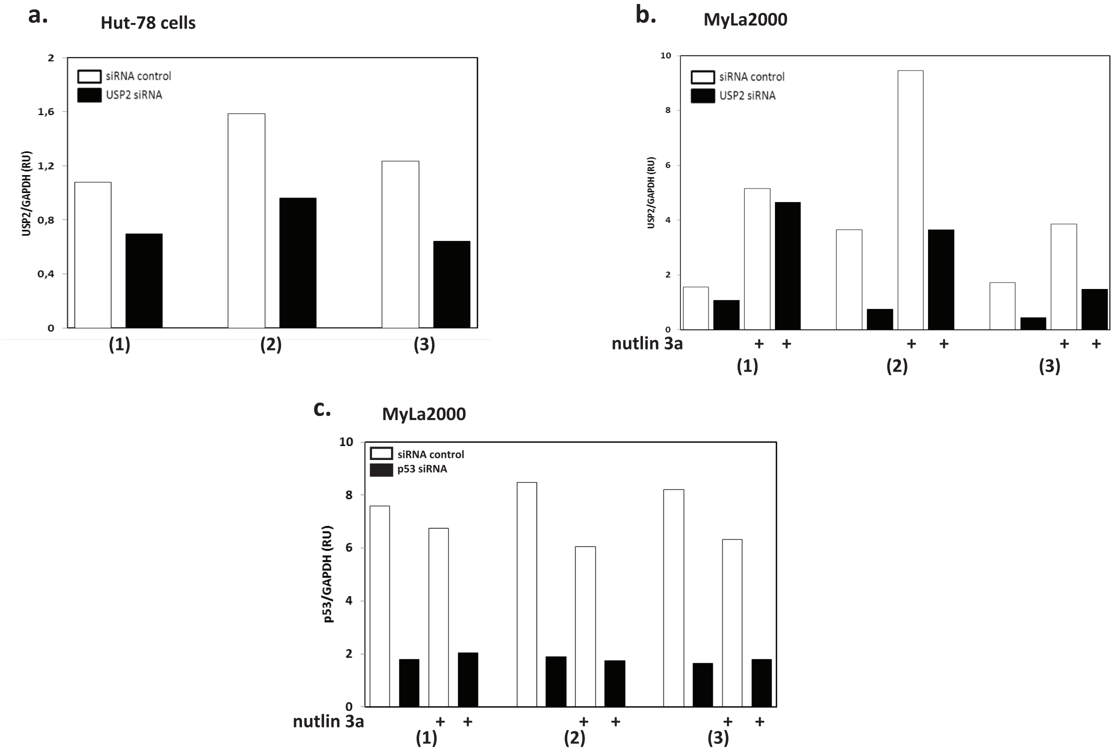

Supplementary Figure S1: Evaluation of USP2 and p53 knockdown efficiency in MyLa2000 and Hut-78 by quantitative RT-PCR. USP2 **a, b.** and p53 **c.** mRNA level were quantified in Hut-78 (a) or MyLa2000 cells (b, c) transfected with siRNA control or USP2 siRNA. b-c) MyLa2000 cells were exposed to 5μM nutlin3a for 24h after transfection. Data was normalized to GAPDH and expressed as relative units (RU). Results from 3 independent repeats were shown.

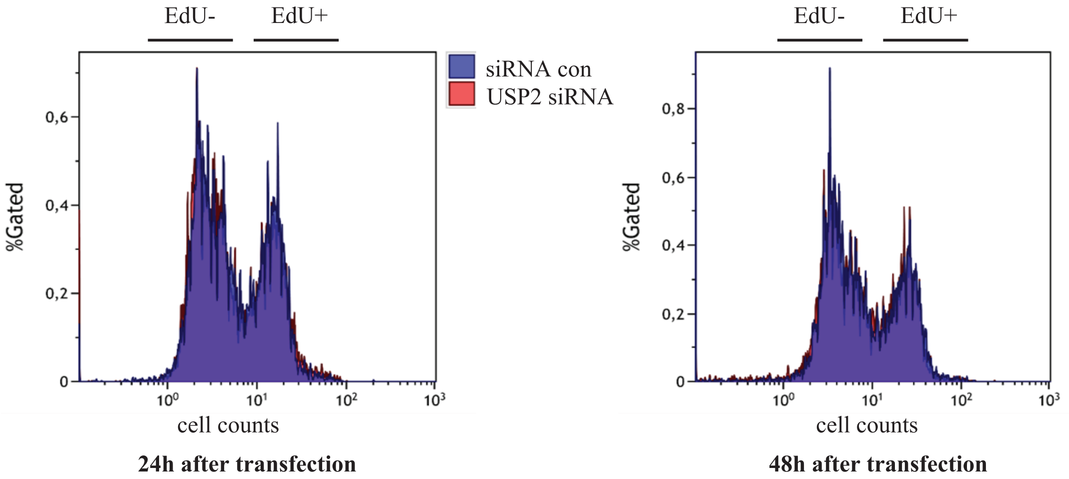

Supplementary Figure S2: USP2 knockdown does not influence MyLa2000 cells proliferation. 50 nM siRNA was used for the specific knockdown of USP2. 24h and 48h after transfection, EdU was added at a 10 µM concentration to the transfected cells 40 min before harvesting. A Click-iT EdU Alexa Fluor 488 Flow Cytometry Assay Kit was used according to manufacturer's instructions and analysed by flow cytometry to determine EdU positive cells.

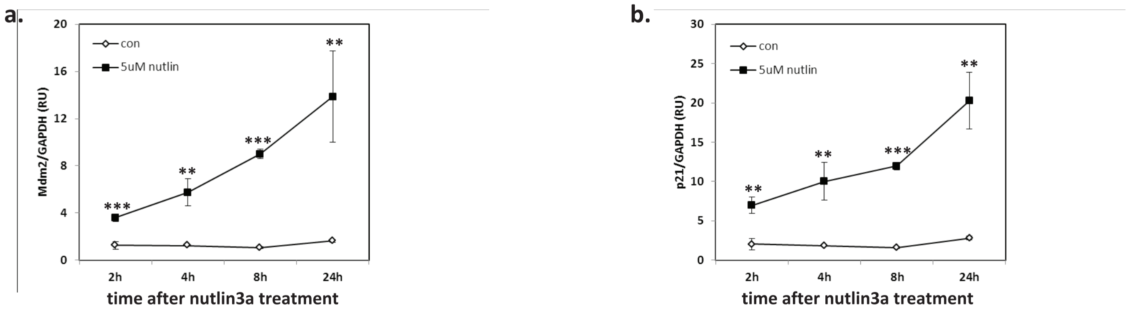

Supplementary Figure S3: The kinetics of Mdm2 and p21 expression in MyLa2000 upon nutlin3a treatment. $p53^{wt}$ CTCL cell line, MyLa2000, was subjected to 5µM nutlin3a **a-b.** as shown in Methods. The expression of Mdm2 (a) and p21 (b) was measured by qPCR 2h-24h after the treatments. Data was normalized to GAPDH and expressed as relative units (RU). The experiments were repeated 3 times. Unpaired T test was used to calculate P-value, error bars, SD, **, P<0.01; ***, P<0.001.
